# TF-TAVI and SAVR post-procedural complications in Germany 2021/2022 – impact on healthcare resource consumption

**DOI:** 10.1007/s10198-025-01838-8

**Published:** 2025-10-14

**Authors:** Alexander Maier, Alicja Zientara, Markus Jäckel, Jonathan Rilinger, Christian Weber, Vera Oettinger, Lukas A. Heger, Derek Hazard, Roman Gottardi, Martin Czerny, Dirk Westermann, Constantin von zur Mühlen, Klaus Kaier

**Affiliations:** 1 Department of Cardiology and Angiology, University Heart Center Freiburg – Bad Krozingen, Faculty of Medicine, University of Freiburg, Freiburg, Germany; 2 Department of Cardiovascular Surgery, University Heart Center Freiburg – Bad Krozingen, Faculty of Medicine, University of Freiburg, Freiburg, Germany; 3 Institute of Medical Biometry and Statistics, Faculty of Medicine, University of Freiburg, Freiburg, Germany

**Keywords:** TAVI, SAVR, Complications, Healthcare resources

## Abstract

**Background:**

Post-procedural complications after transfemoral transcatheter aortic valve implantation (TF-TAVI) lead to extended healthcare resource consumption. A comparison with resource consumption after surgical aortic stenosis valve replacement (SAVR) complications has not been conducted.

**Methods:**

The impact of acute kidney injury (AKI), stroke, severe bleeding and permanent pacemaker implantation (PPI) on length of stay, mechanical ventilation > 48 h and reimbursement was analyzed by risk-adjusted linear and logistic regression analyses of all German aortic stenosis TF-TAVI and SAVR cases 2021/2022.

**Results:**

48,565 TF-TAVI and 9,803 SAVR procedures for aortic stenosis treatment were performed in Germany 2021/2022. The length of stay for TF-TAVI was shorter (10.16 ± 7.19 vs. 13.91 ± 9.82 days, p < 0.001), the rate of mechanical ventilation > 48 h was lower after TF-TAVI (1.3% vs. 7.0%, p < 0.001) and reimbursement was higher for TF-TAVI (26,483 ± 4,487 vs. 20,538 ± 11,748 €, p < 0.001). Length of stay was increased by all investigated complications after TF-TAVI and SAVR (p < 0.001) with the highest increase after bleeding in TF-TAVI. Odds ratios for mechanical ventilation > 48 h were significantly increased for stroke, severe bleeding and AKI (p < 0.001) but not for PPI after both TF-TAVI and SAVR with the highest OR increase after bleeding in TF-TAVI. Reimbursement was significantly increased after TF-TAVI and SAVR by all investigated complications (p < 0.001) finding significantly higher increases after SAVR compared to TF-TAVI for all complications.

The total hospital stay after stroke, AKI and PPM was longer for SAVR (p < 0.001), while severe bleeding led to longer total hospital stay after TF-TAVI (p < 0.001). Total reimbursement remained higher for TF-TAVI after all investigated complications (p < 0.001).

**Conclusion:**

Healthcare resource consumption differs between TF-TAVI and SAVR for aortic stenosis treatment also after procedural complications. SAVR is associated with longer hospitalization and more mechanical ventilation, while TF-TAVI is associated with higher reimbursement in the German healthcare system. Complications lead to increased resource use for both procedures with higher extra reimbursement for SAVR and more extra hospital days for TF-TAVI after bleeding reversing the length of stay advantage.

## Introduction

Cardiovascular diseases are among the greatest health and economic burdens for Western societies and their healthcare systems [1]. Aortic stenosis is of particular relevance as it is common in the aging Western population and expensive to treat [2–4]. Transcatheter aortic valve implantation (TAVI) and surgical aortic valve replacement (SAVR) are at the forefront of guideline-based therapeutic options for severe aortic stenosis. The decision on which therapeutic intervention benefits a patient the most is multifactorial and recommended by a heart team, with age and surgical risk being among the most decisive factors [5].

In addition to purely medical aspects, economic considerations for efficient resource use are becoming increasingly important in evaluating therapeutic interventions. This also applies to the treatment of aortic valve stenosis. When TAVI was introduced into clinical practice for high-risk surgical patients in the 2000 s, prosthesis costs were high, and the procedure was complex. This made the procedure expensive compared to its surgical counterpart, SAVR [6, 7]. However, in recent years, the costs of TAVI prostheses and procedures have decreased and cost-effectiveness became more favorable [8, 9]. Given the expected increase in patient numbers with severe aortic stenosis and the associated high therapy needs, understanding the cost structure of both procedures is crucial for affordable and high-quality medical care.

In Germany, it has been shown that periprocedural bleeding complications, acute kidney injury (AKI), and strokes associated with TAVI lead to increased resource use and higher reimbursement rates in 2013 [10]. However, the impact of these highly relevant post-procedural complications after TAVI and SAVR on resource use and their consequences within the German reimbursement system in recent years have not been compared yet [11]. Understanding these impacts is interesting for an efficient utilization of limited healthcare resources.

Therefore, this study investigates and compares the influence of the complications bleeding, AKI, strokes and permanent pacemaker implantation (PPI) following transfemoral TAVI (TF-TAVI) and SAVR for aortic valve stenosis treatment on hospital length of stay, need for mechanical ventilation, and reimbursement. The analysis is based on data obtained from the German Federal Statistical Office, which provides access to anonymized, aggregated inpatient billing data from all German hospitals through its research data center. By examining the economic dimensions of aortic valve stenosis therapy, this analysis aims to enhance the understanding of resource utilization and reimbursement associated with TAVI and SAVR.

## Methods

### Data source

German hospitals must report all cases including interventional treatments and surgeries to InEK GmbH using ICD-10-GM and OPS codes. Since 2004, the Federal Statistical Office (DeStatis) has collected and archived data on all hospital stays, including diagnoses, comorbidities, and procedures, via these codes. Data has been accessible for research through DeStatis'Research Data Center since 2005. Data requests from DeStatis do not require patient consent or ethics committee approval, as information is anonymized and non-traceable. Groups with fewer than three events are excluded to maintain anonymity.

The analysis included all isolated TF-TAVI and SAVR procedures performed on patients with aortic valve stenosis (I35.0/I06.0) or combined aortic valve disease (stenosis and insufficiency, I35.2/I06.2) in Germany during 2021 and 2022. Cases treated solely for aortic valve insufficiency (without stenosis, I35.0, I35.2, I06.0, I06.2) were excluded from the analysis. Additionally, patients who underwent a percutaneous coronary intervention (8–837.*) or another heart surgery (5–361.*, 5–362.*, 5–363.*, 5–351.1*, 5–351.2*, 5–353.1, 5–353.2, 5–351.4*, 5–353.4, 5–353.5) during the same hospital stay were not included.

In previous studies, Reinöhl et al. identified 20 baseline patient characteristics to describe risk profiles between procedural groups [12]. ICD/OPS-codes for patient baseline characteristics and resource usage (length of stay, need for mechanical ventilation > 48 h, and reimbursement) are given elsewhere [13–15].

Requested complications were stroke (I63.*), severe bleeding (transfusion of more than five red blood cell concentrates, 8–800.c1–8–800.cr), acute kidney injury (N17.*) and permanent pacemaker implantation (5–337.*). Patients without complications were defined and requested as cases without stroke, bleeding, AKI and PPI.

### Statistical methods

Categorical variables were presented as counts (n) and percentages (%). Continuous variables were presented as means with standard deviations. Comparisons between the TF-TAVI and the SAVR group were made using Pearson's Chi^2^ test for categorical variables and two-sided unpaired t-tests for continuous variables. Odds ratios were compared with the Z-test. To assess the impact of post-procedural complications on selected healthcare resources, unadjusted linear and logistic regression analyses were initially conducted. Logistic regression analyses were used for dichotomous endpoints, and linear regression analyses were applied for continuous endpoints. In order to control for varying patient profiles, multivariable logistic or linear regression models were repeated in a final step, utilizing all baseline patient characteristics from Table 1 as potential confounders. In all regression models, cluster robust standard errors at the hospital level were specified to accommodate the correlation of error terms among patients treated at the same hospital. Statistical significance was considered as p-value < 0.05. No adjustments for multiple testing were done. All analyses were carried out using Stata 18 (StataCorp, College Station, Texas, USA).

**Table 1:** Patients baseline characteristics. SD: standard deviation, NYHA: New York Heart Association, MI: myocardial infarction, CABG: coronary artery bypass grafting, COPD: chronic obstructive pulmonary disease, CKD: chronic kidney disease, GFR: glomerular filtration rate. Two-tailed t-test or chi^2^-test as appropriate

	**Total**	**TF-TAVI**	**SAVR**	**p**
N	58,368 (100.0%)	48,565 (83.2%)	9,803 (16.8%)	
2021	27,978 (47.9%)	23,292 (48.0%)	4,686 (47.8%)	
2022	30,390 (52.1%)	25,273 (52.0%)	5,117 (52.2%)	
Emergency Administration	5,560 (9.5%)	4,850 (10.0%)	710 (7.2%)	< 0.001
Age (± SD)	78.23 ± 9.23	81.03 ± 6.26	64.35 ± 9.02	< 0.001
Female sex	26,190 (44.9%)	22,961 (47.3%)	3,229 (32.9%)	< 0.001
Logistic EuroSCORE [%]	11.21 ± 9.35	12.69 ± 9.44	3.92 ± 3.81	< 0.001
NYHA III/IV	26,230 (44.9%)	23,417 (48.2%)	2,813 (28.7%)	< 0.001
Arterial hypertension	36,804 (63.1%)	30,886 (63.6%)	5,918 (60.4%)	< 0.001
Atrial fibrillation	25,474 (43.6%)	21,312 (43.9%)	4,162 (42.5%)	0.009
Diabetes mellitus	17,071 (29.2%)	14,832 (30.5%)	2,239 (22.8%)	< 0.001
Coronary artery disease	26,663 (45.7%)	24,740 (50.9%)	1,923 (19.6%)	< 0.001
Previous MI within 4 months	805 (1.4%)	725 (1.5%)	80 (0.8%)	< 0.001
Previous MI within 1 year	336 (0.6%)	295 (0.6%)	41 (0.4%)	0.024
Previous MI within more than 1 year	1,988 (3.4%)	1,819 (3.7%)	169 (1.7%)	< 0.001
Previous CABG	3,537 (6.1%)	3,451 (7.1%)	86 (0.9%)	< 0.001
Previous cardiac surgery	6,799 (11.6%)	6,323 (13.0%)	476 (4.9%)	< 0.001
Atherosclerotic disease	4,684 (8.0%)	4,313 (8.9%)	371 (3.8%)	< 0.001
Carotid disease	3,205 (5.5%)	2,844 (5.9%)	361 (3.7%)	< 0.001
COPD	4,921 (8.4%)	4,196 (8.6%)	725 (7.4%)	< 0.001
Pulmonary Hypertension	8,863 (15.2%)	8,133 (16.7%)	730 (7.4%)	< 0.001
CKD, GFR < 15 ml/min/1,73 m^2^	1,114 (1.9%)	1,021 (2.1%)	93 (0.9%)	< 0.001
CKD, GFR < 30 ml/min/1,73 m^2^	1,828 (3.1%)	1,770 (3.6%)	58 (0.6%)	< 0.001

## Results

### Patient characteristics, complication rates and resource usage

A total of 48,565 TF-TAVI and 9,803 SAVR operations were performed for aortic stenosis treatment in 2021 and 2022.. Patients baseline characteristics are given in Table 1. TF-TAVI patients were older (81.03 ± 6.26 vs. 64.35 ± 9.02 years, p < 0.001) and more likely to be female (47.3% vs. 32.9%, p < 0.001). All requested comorbidities were significantly more frequent in the TF-TAVI group. Consequently, the logistic EuroSCORE was higher in the TF-TAVI group (12.69 ± 9.44% vs. 3.92 ± 3.81%, p < 0.001).

We further requested the four major complications after TF-TAVI and SAVR, which are stroke, severe bleeding, AKI and PPI [11]. Stroke rates were the same for both groups (2.1% each). Severe bleeding, which was defined as the need for more than five red blood cell concentrates transfusion, was observed more often in the SAVR group (9.0% vs. 1.7%, p < 0.001). AKI also occurred significantly more often in the SAVR group (9.8% vs. 8.6%, p < 0.001), while PPI was necessary more often in the TF-TAVI group (12.7% vs. 4.6%, p < 0.001). The complication rates are given in Table 2.

**Table 2:** Complication rates after TF-TAVI and SAVR. RBC: red blood cell. Chi^2^-test

	**Total** n = 58,368	**TF-TAVI** n = 48,565	**SAVR** n = 9,803	**p**
Stroke	1,241 (2.1%)	1,035 (2.1%)	206 (2.1%)	0.852
Severe bleeding (> 5 RBC concentrates)	1,721 (2.9%)	841 (1.7%)	*880 (9.0%)*	< 0.001
Acute kidney injury	5,150 (8.8%)	4,188 (8.6%)	*962 (9.8%)*	< 0.001
Permanent pacemaker	6,613 (11.3%)	*6,158 (12.7%)*	455 (4.6%)	< 0.001

The requested healthcare resources were hospital length of stay, mechanical ventilation > 48 h and reimbursement (Table 3). SAVR patients had a longer length of stay (10.16 ± 7.19 vs. 13.91 ± 9.82 days, p > 0.001) and were more likely for a mechanical ventilation > 48 h (7.0% vs. 1.3%, p < 0.001). In contrast, reimbursement was significantly higher for TF-TAVI (26,483 ± 4,487 € vs. 20,538 ± 11,748 €, p < 0.001).

**Table 3:** Resource usage after TAVI and SAVR. SD: standard deviation. Two-tailed t-test or chi^2^-test as appropriate

	**Total** n = 58,368	**TF-TAVI** n = 48,565	**SAVR** n = 9,803	**p**
Length of stay [days] (± SD)	10.79 ± 7.82	*10.16* ± *7.19*	13.91 ± 9.82	< 0.001
Mechanical ventilation > 48 h	1,338 (2.3%)	*655 (1,3%)*	683 (7.0%)	< 0.001
Reimbursement [€] (± SD)	25,485.07 ± 6,698.93	26,483.64 ± 4,487.71	*20,538.06* ± *11,748.17*	< 0.001

### Impact of complications on additional healthcare resource usage

The impact of complications on healthcare resource usage after TF-TAVI and SAVR was analyzed using univariate and multivariate linear and logistic regression models. For risk-adjustment patients baseline characteristics from Table 1 were taken into account.

First, the impact of complications on additional length of stay was analyzed. Stroke, bleeding, AKI and PPI after TF-TAVI was associated both unadjusted and risk-adjusted with a significantly longer length of hospital stay between 2.5 days for permanent PPI and 13 days for severe bleeding (Table 4A and 4B). In parallel, all investigated complications were observed with a significantly increased length of stay between 3.9 and 10.4 days after SAVR (Table 5A and 5B).

**Table 4:** Impact of complications on TF-TAVI length of stay. (**A**) unadjusted extra length of stay, univariate linear regression analysis. (**B**) Baseline characteristics risk-adjusted extra length of stay, multivariate linear regression analysis

TF-TAVI Extra length of stay	unadjusted			
	Coefficient	p	95% CI	
Stroke	5.56	< 0.001	4.50	6.62
Severe bleeding (> 5 RBC concentrates)	13.73	< 0.001	12.08	15.37
Acute kidney injury	6.40	< 0.001	5.70	7.11
Permanent pacemaker	2.58	< 0.001	2.24	2.92
**TF-TAVI** **Extra length of stay**	**risk-adjusted**			
	Coefficient	p	95% CI	
Stroke	5.08	< 0.001	4.08	6.08
Severe bleeding (> 5 RBC concentrates)	12.92	< 0.001	11.30	14.54
Acute kidney injury	5.28	< 0.001	4.62	5.94
Permanent pacemaker	2.54	< 0.001	2.23	2.86

**Table 5:** Impact of complications on SAVR length of stay. (**A**) unadjusted extra length of stay, univariate linear regression analysis. (**B**) Baseline characteristics risk-adjusted extra length of stay, multivariate linear regression analysis

SAVR Extra length of stay	unadjusted			
	Coefficient	p	95% CI	
Stroke	6.71	< 0.001	3.98	9.43
Severe bleeding (> 5 RBC concentrates)	10.37	< 0.001	8.54	12.19
Acute kidney injury	6.88	< 0.001	5.43	8.33
Permanent pacemaker	4.12	< 0.001	2.78	5.45
**SAVR** **Extra length of stay**	**risk-adjusted**			
	Coefficient	P	95% CI	
Stroke	6.28	< 0.001	3.66	8.91
Severe bleeding (> 5 RBC concentrates)	9.00	< 0.001	7.38	10.61
Acute kidney injury	5.78	< 0.001	4.42	7.13
Permanent pacemaker	3.90	< 0.001	2.55	5.25

We further analyzed the impact of complications on the odds ratios of the need for mechanical ventilation longer than 48 h. Stroke, severe bleeding and AKI significantly increased the unadjusted and risk-adjusted likelihood for mechanical ventilation > 48 h for TF-TAVI and SAVR. In contrast, PPI did not significantly increase the odds ratio for mechanical ventilation > 48 h for both TF-TAVI and SAVR (Table 6A, 6B, 7A and 7B).

**Table 6:** Impact of complications on mechanical ventilation > 48 h after TF-TAVI. (**A**) unadjusted odds, univariate logistic regression analysis. (**B**) Baseline characteristics risk-adjusted odds ratio, multivariate logistic regression analysis

TF-TAVI Odds ratio Mechanical ventilation > 48 h	unadjusted			
	Odds ratio	p	95% CI	
Stroke	6.81	< 0.001	4.83	9.59
Severe bleeding (> 5 RBC concentrates)	37.90	< 0.001	30.12	47.71
Acute kidney injury	8.08	< 0.001	6.62	9.86
Permanent pacemaker	1.19	0.190	0.92	1.56
**TF-TAVI Odds ratio** **Mechanical ventilation > 48 h**	**risk-adjusted**			
	Odds ratio	p	95% CI	
Stroke	6.38	< 0.001	4.41	9.22
Severe bleeding (> 5 RBC concentrates)	32.48	< 0.001	25.29	41.70
Acute kidney injury	7.78	< 0.001	6.18	9.78
Permanent pacemaker	1.20	0.167	0.93	1.55

**Table 7:** Impact of complications on mechanical > 48 h ventilation after SAVR. (**A**) unadjusted odds, univariate logistic regression analysis. (**B**) Baseline characteristics risk-adjusted odds ratio, multivariate logistic regression analysis

SAVR Odds ratio Mechanical ventilation > 48 h	unadjusted			
	Odds ratio	p	95% CI	
Stroke	9.27	< 0.001	6.48	13.25
Severe bleeding (> 5 RBC concentrates)	14.76	< 0.001	11.82	18.43
Acute kidney injury	6.09	< 0.001	4.93	7.51
Permanent pacemaker	1.51	0.097	0.93	2.45
**SAVR Odds ratio** **Mechanical ventilation > 48 h**	**risk-adjusted**			
	Odds ratio	p	95% CI	
Stroke	9.49	< 0.001	6.72	13.41
Severe bleeding (> 5 RBC concentrates)	13.42	< 0.001	10.77	16.72
Acute kidney injury	5.13	< 0.001	4.11	6.42
Permanent pacemaker	1.50	0.099	0.93	2.43

Lastly, the impact of complications on extra reimbursement was investigated. For both TF-TAVI and SAVR all investigated complications were associated with a significantly higher reimbursement before and after risk-adjustment for patient characteristics in the German DRG system (Table 8A, 8B, 9A and 9B).

**Table 8:** Impact of complications on TF-TAVI reimbursement. (**A**) unadjusted extra reimbursement, univariate linear regression analysis. (**B**) Baseline characteristics risk-adjusted extra reimbursement, multivariate linear regression analysis

TF-TAVI – Extra reimbursement [€]	unadjusted			
	Coefficient	p	95% CI	
Stroke	2,132.07	< 0.001	1,422.70	2,841.45
Severe bleeding (> 5 RBC concentrates)	13,280.88	< 0.001	11,477.56	15,084.20
Acute kidney injury	2,284.58	< 0.001	1,888.23	2,680.93
Permanent pacemaker	304.44	< 0.001	168.10	440.78
**TF-TAVI – Extra reimbursement [€]**	**risk-adjusted**			
	Coefficient	p	95% CI	
Stroke	2,038.91	< 0.001	1,329.79	2,748.02
Severe bleeding (> 5 RBC concentrates)	13,037.57	< 0.001	11,245.62	14,829.52
Acute kidney injury	2,153.11	< 0.001	1,773.86	2,532.37
Permanent pacemaker	299.24	< 0.001	165.70	432.77

**Table 9:** Impact of complications on SAVR reimbursement. (**A**) unadjusted extra reimbursement, univariate linear regression analysis. (**B**) Baseline characteristics risk-adjusted extra reimbursement, multivariate linear regression analysis

SAVR – Extra reimbursement [€]	unadjusted			
	Coefficient	p	95% CI	
Stroke	8,607.35	< 0.001	5,316.26	11,898.45
Severe bleeding (> 5 RBC concentrates)	16,591.04	< 0.001	14,272.49	18,909.59
Acute kidney injury	9,069.15	< 0.001	7,040.95	11,097.35
Permanent pacemaker	4,147.79	< 0.001	2,535.64	5,759.93
**SAVR – Extra reimbursement [€]**	**risk-adjusted**			
	Coefficient	p	95% CI	
Stroke	8,358.46	< 0.001	5,170.62	11,546.30
Severe bleeding (> 5 RBC concentrates)	15,606.54	< 0.001	13,492.44	17,720.63
Acute kidney injury	8,470.28	< 0.001	6,544.82	10,395.75
Permanent pacemaker	4,090.56	< 0.001	2,435.80	5,745.33

### Comparison of additional healthcare resource usage after complications

**Fig. 1:**
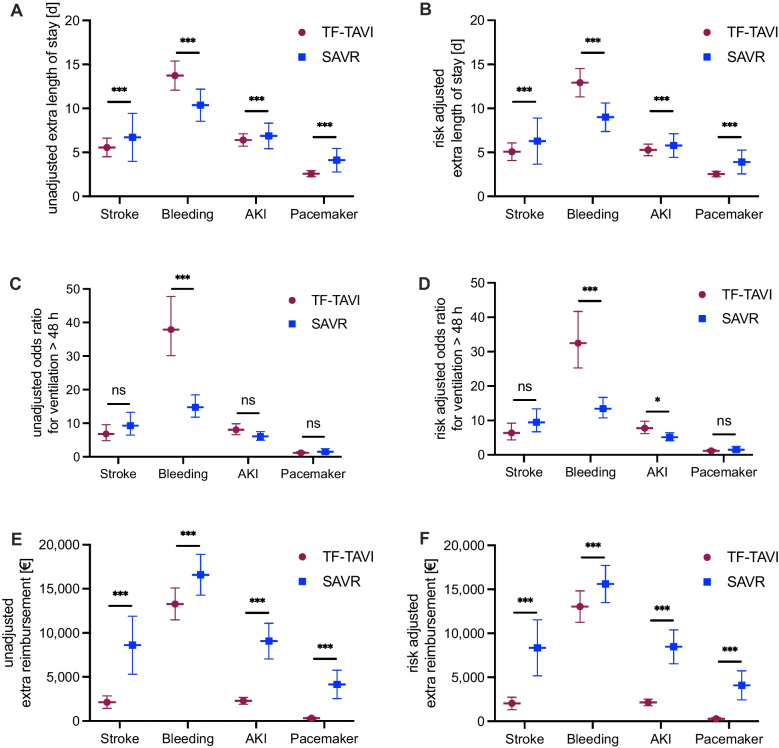
Comparison of additional healthcare resource usage after complications. A. Comparison of risk unadjusted extra length of hospital stay after complications between TF-TAVI and SAVR. **B.** Comparison of risk adjusted extra length of hospital stay after complications between TF-TAVI and SAVR. **C.** Comparison of risk unadjusted odds ratios of the need for mechanical ventilation > 48 h after complications between TF-TAVI and SAVR. **D.** Comparison of risk adjusted odds ratios of the need for mechanical ventilation > 48 h after complications between TF-TAVI and SAVR. **E.** Comparison of risk unadjusted extra reimbursement after complications between TF-TAVI and SAVR. **F.** Comparison of risk adjusted extra reimbursement after complications between TF-TAVI and SAVR. Bleeding was defined as transfusion of more than five red blood cell concentrates. TF-TAVI: transfemoral transcatheter aortic valve implantation, SAVR: surgical aortic valve replacement. AKI: Acute kidney injury. Shown are coefficients with 95% CI and odds ratios with 95% CI, which are given in Tables 4, 5, 6, 7, 8 and 9. Unpaired two-tailed t-test for continuous variables or Z-test for odds ratios. *** indicates p < 0.001. * indicates p < 0.05. ns indicates no significant difference

The extra length of hospital stay after stroke was significantly longer after SAVR compared to TF-TAVI both unadjusted (5.56 ± 0.54 vs. 6.71 ± 1.39 extra days, p < 0.001) and risk adjusted (5.08 ± 0.51 vs. 6.28 ± 1.34 extra days, p < 0.001). Severe bleeding induced significantly more extra hospital days after TF-TAVI (13.73 ± 0.84 vs. 10.37 ± 0.93 extra days, p < 0.001), which was consistent after risk adjustment (12.92 ± 0.83 vs. 9.00 ± 0.83, p < 0.001). AKI was linked to significantly more extra days after SAVR compared to TF-TAVI (6.40 ± 0.36 vs. 6.88 ± 0.74 extra days, p < 0.001), which again was consistent after risk adjustment (5.28 ± 0.34 vs. 5.78 ± 0.69 extra days, p < 0.001). PPI was associated with significant more extra hospital days after SAVR compared to TF-TAVI (2.58 ± 0.17 vs. 4.12 ± 0.68 extra days, p < 0.001), again consistent after risk adjustment (2.54 ± 0.16 vs. 3.90 ± 0.69 extra days, p < 0.001, Fig. 1A and 1B).

The risk of mechanical ventilation > 48 h after complications showed diverse results (Fig. 1C and 1D). While a stroke was not connected to significant both unadjusted and risk adjusted odds ratio differences for mechanical ventilation > 48 h, bleeding was associated with a significant higher odds ratio in the TF-TAVI cohort compared to the SAVR cohort both unadjusted (37.90; 95% CI: 30.12–47.71 vs. 14.76 95; % CI: 11.82–18.43, p < 0.001) and risk adjusted (32.48; 95% CI: 25.29–41.70 vs. 13.42 95% CI: 10.77–16.72, p < 0.001). The unadjusted odds ratio for ventilation > 48 h after AKI did not differ significantly. After risk adjustment the odds ratio for AKI was significantly higher for the SAVR cohort (5.13 95% CI: 4.11–6.42 vs. 7.78; 95% CI: 6.18–9.78, p < 0.05). The odds ratios for mechanical ventilation > 48 h after PPI did not differ significantly between TF-TAVI and SAVR.

The additional reimbursement was consistently higher after SAVR for the investigated complications both unadjusted and risk adjusted (Fig. 1E and 1F). In detail, a stroke induced an additional unadjusted reimbursement of 2,132.07 ± 361.93 € for TF-TAVI versus 8,607.35 ± 1,679.13 € for SAVR (p < 0.001). The risk adjusted extra reimbursement after stroke was 2,038.91 ± 361.79 € for TF-TAVI and 8,358.46 ± 1,626.45 € for SAVR (p < 0.001). Bleeding was associated with an additional unadjusted reimbursement of 13,280.88 ± 920.06 € for TF-TAVI versus 16,591.04 ± 1,182.93 € for SAVR (p < 0.001). The risk adjusted extra reimbursement after stroke was 13,037.57 ± 914.26 € in the TF-TAVI cohort versus 15,606.54 ± 1,078.62 € in the SAVR group (p < 0.001). AKI after TF-TAVI was associated with an additional unadjusted reimbursement of 2,284.58 ± 202.22 € and 9,069.15 ± 1,034.80 € after SAVR (p < 0.001). The risk adjusted extra reimbursement after AKI was 2,153.11 ± 193.50 € after TF-TAVI versus 8,470.28 ± 982.38 € after SAVR (p < 0.001). Finally, PPI was connected with an additional unadjusted reimbursement of 304.44 ± 69.56 € after TF-TAVI versus 4,147.79 ± 822.52 € after SAVR (p < 0.001). The risk adjusted extra reimbursement after PPI was 299.24 ± 68.13 € (TF-TAVI) vs. 4,090.56 ± 844.27 € (SAVR, p < 0.001).

### Comparison of total healthcare resource usage after complications

**Fig. 2:**
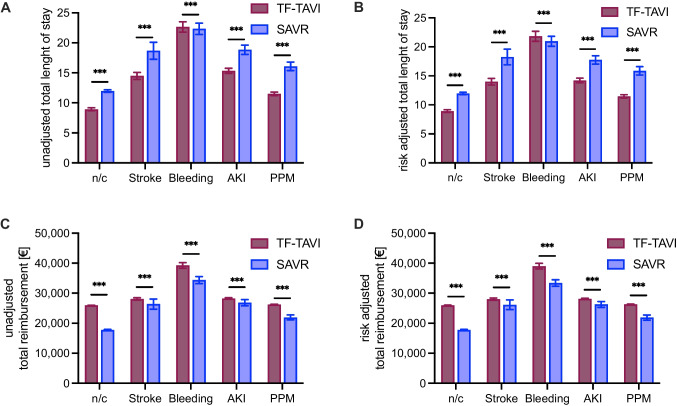
Comparison of total healthcare resource usage after complications. A. Unadjusted total length of stay with and without complications after TF-TAVI and SAVR. n/c (TF-TAVI 8.93 ± 0.24 vs. SAVR 11.97 ± 0.21 days), stroke (TF-TAVI 14.49 ± 0.59 vs. SAVR 18.68 ± 1.41 days), bleeding (TF-TAVI 22.65 ± 0.87 vs. SAVR 22.34 ± 0.95 days), AKI (TF-TAVI 15.33 ± 0.43 vs. SAVR 18.85 ± 0.77 days), PPM (TF-TAVI 11.51 ± 0.30 vs. SAVR 16.09 ± 0.71 days). **B.** Risk-adjusted total length of stay with and without complications after TF-TAVI and SAVR. n/c (TF-TAVI 8.93 ± 0.24 vs. SAVR 11.97 ± 0.21 days), stroke (TF-TAVI 14.00 ± 0.56 vs. SAVR 18.26 ± 1.36 days), bleeding (TF-TAVI 21.84 ± 0.86 vs. SAVR 20.97 ± 0.85 days), AKI (TF-TAVI 14.21 ± 0.41 vs. SAVR 17.75 ± 0.72 days), PPM (TF-TAVI 11.47 ± 0.29 vs. SAVR 15.87 ± 0.72 days). **C.** Unadjusted total reimbursement with and without complications after TF-TAVI and SAVR. n/c (TF-TAVI 25,972.61 ± 51.78 vs. SAVR 17,785.33 ± 134.74 €), stroke (TF-TAVI 28.104,68 ± 365.61 vs. SAVR 26,392.68 ± 1,684.53 €), bleeding (TF-TAVI 39,253.49 ± 921.52 vs. SAVR 34,376.37 ± 1,190.58 €), AKI (TF-TAVI 28,257.19 ± 208.74 vs. SAVR 26,854.48 ± 1,043.53 €), PPM (TF-TAVI 26,277.05 ± 86.72 vs. SAVR 21,933.12 ± 833.49 €). **D.** Risk-adjusted total reimbursement with and without complications after TF-TAVI and SAVR. n/c (TF-TAVI 25,972.61 ± 51.78 vs. SAVR 17,785.33 ± 134.74 €), stroke (TF-TAVI 28,011.52 ± 365.48 vs. SAVR 26,143.79 ± 1,632.02 €), bleeding (TF-TAVI 39,010.18 ± 915.73 vs. SAVR 33,391.87 ± 1,087.00 €), AKI (TF-TAVI 28,125.72 ± 200.31 vs. SAVR 26,255.61 ± 991.58 €), PPM (TF-TAVI 26,271.85 ± 85.57 vs. SAVR 21,875.89 ± 854.95 €). TF-TAVI: transfemoral transcatheter aortic valve implantation, SAVR: surgical aortic valve replacement. n/c: no complications, AKI: acute kidney injury, PPM: permanent pacemaker implantation. Data are shown as mean ± standard deviation. Unpaired two-tailed t-test for continuous variables.*** indicates p < 0.001

Finally, we compared total healthcare resource usage after complications for TF-TAVI and SAVR. Patients with an uncomplicated TF-TAVI procedure had a total hospital length of stay of 8.93 ± 0.24 days, patients with an uncomplicated SAVR had a significantly longer total hospital length of stay of 11.97 ± 0.21 days (p < 0.001). This difference remained consistent for stroke, AKI and PPI, but turned into a significant longer total length of stay through bleeding for TF-TAVI compared to SAVR (p < 0.001, Fig. 2A and 2B).

Total reimbursement after an uncomplicated TF-TAVI was 25,972.61 ± 51.78 € compared to 17,785.33 ± 134.74 € after uncomplicated SAVR (p < 0.001). This significantly higher reimbursement after TF-TAVI remained consistent after all investigated complications (p < 0.001, Fig. 2C and 2D).

## Discussion

This study, based on data from the Federal Statistical Office for the years 2021 and 2022, showed that the consumption of healthcare resources – measured by hospital length of stay, ventilation time over 48 h, and hospital reimbursement following TF-TAVI and SAVR – significantly increased due to peri- and post-procedural complications such as stroke, major bleeding, and acute kidney failure. In the case of PPI as a complication, resource consumption, in terms of hospital stay and reimbursement, increased significantly following both TF-TAVI and SAVR. PPI did not significantly impact the likelihood of ventilation time exceeding 48 h for both procedures. Additionally, the study revealed that the average total length of hospital stay after SAVR, both with and without complications, was longer compared to TF-TAVI with the exception of severe bleeding as a complication. Despite a higher increase in reimbursement following complications, SAVR remains the treatment associated with lower overall reimbursement compared to TAVI.

The number of TF-TAVI procedures in Germany has risen significantly since 2013, increasing from 9,147 in 2013 to 25,273 in 2022. Over this period, patient characteristics have shifted. While the average age remained stable at around 81 years, the EuroSCORE dropped from 22% in 2013 to 12—13% in 2022 [10]. Comorbidities such as prior heart surgeries, COPD, atrial fibrillation, diabetes, and atherosclerosis have also decreased, while severe renal insufficiency remained constant. Conversely, surgical aortic valve replacements for aortic stenosis have declined, from 6,226 in 2015 to 5,111 in 2022 [16]. The average age of surgical patients has decreased from 68 to 64 years, and their EuroSCORE dropped slightly from five to four [16]. Other conditions, such as atrial fibrillation and severe renal insufficiency, remained stable or showed minor declines. These trends reflect a shift in the risk profile of TAVI and surgical patients, with TAVI increasingly used for patients with moderate or even lower surgical risk, influenced by stronger evidence supporting TAVI in lower-risk groups and patient preference for minimally invasive procedures [17]. This change has led to a slightly lower-risk surgical population and a reduction in surgical procedures overall, while the technical developments like minimally invasive surgical implantations in many places became the standard of care. The ongoing expansion of TAVI indications, including trials investigating its use in moderate aortic stenosis, suggests that TAVI number increases and patient risk profile shifts are likely to continue in the future.

From 2013 to 2022, relevant shifts in complication rates for TF-TAVI (transcatheter aortic valve implantation) and surgical aortic valve replacement (SAVR) were observed. For TF-TAVI stroke rates decreased from 2.57% in 2013 to 2.1% in 2021/2022. Severe bleeding dropped significantly from 6.7% to 1.7%. AKI increased from 5.18% to 8.6% and PPI rates declined from 15.61% to 12.7% [10]. The improvements, particularly the reduction in bleeding, are attributed to healthier patient profiles, technological advances (e.g., smaller catheters, improved vascular closure systems), improved perioperative management, and tailored anticoagulation protocols. The rise in AKI might result from stricter documentation practices. For SAVR stroke rates remained stable at 1.6—2.1% between 2014 and 2022. Bleeding rates were steady at ~ 9%, AKI rose from 5.3% to 9.8% and PPI rates increased slightly from 3.9% to 4.6% [18]. While SAVR complications have mostly remained stable, the growing experience with TF-TAVI has resulted in a notable decrease in its complication rates, driven partly by a healthier patient profile. Conversely, despite improvements in patient selection, SAVR complication rates have not significantly declined, suggesting the procedure's inherent limitations. TF-TAVI is expected to benefit further from technological advancements, reducing complications and broadening its applicability. Meanwhile, SAVR, despite being a mature procedure, could see incremental improvements due to competitive pressure from TAVI. Enhanced safety and reduced resource use remain crucial goals for both approaches.

The overall resource consumption for aortic valve replacement procedures in Germany has evolved significantly over the years, with TF-TAVI showing remarkable reductions. The average hospital stay decreased from 17.04 days in 2013 to 10.19 days in 2021/2022. The percentage of patients requiring ventilation for over 48 h dropped from 5.27% to 1.3%. Hospital reimbursement costs declined from 34,611 € in 2013 to 26,483 € in 2021/2022 [10]. These improvements highlight TAVI's progression towards a leaner, more patient- and resource-efficient procedure. Contributing factors include healthier, lower-risk patient populations and advancements in technology.

In contrast, average hospital stay for SAVR remained relatively stable, decreasing slightly from 14.8 days (2014—2016) to 13.9 days in 2021/2022 [18]. SAVR ventilation rates > 48 h also showed little change, with rates ranging from 6.6% (women) to 7.4% (men) between 2011—2014, and 7.0% overall in 2021/2022 [19]. Average SAVR reimbursement costs increased marginally from 19,055 € in 2015 to 20,538 € in 2021/2022 [16]. These data reflect SAVR's maturity as a procedure with limited room for economic rationalization.

The reduction in TAVI reimbursement costs over time must be understood within the framework of the German Diagnosis-Related Groups (DRG) system. In 2022, the national base rate ("Bundesbasisfallwert") was 3,833.07 €, compared to 3,156.82 € in 2014 [20]. This base rate, adjusted annually based on legal guidelines, forms the foundation for hospital reimbursement calculations. The reimbursement for a procedure is determined by multiplying the DRG's relative weight by the base rate. For TAVI, the primary DRG code (F98B) had a relative weight of 6.138 in 2022 [21], significantly lower than its 2014 value of 10.553 [22]. SAVR, coded as F03D, also saw its relative weight decline from 5.738 (2014) to 4.633 (2022) [23, 24]. These changes reflect adjustments in cost structures, resource consumption, and procedural efficiencies. TAVI has become less resource-intensive, with shorter hospital stays and reduced ventilation likelihood. Future developments may allow TAVI to be performed on regular wards or even in outpatient settings, further lowering costs. Healthier, lower-risk patients undergoing TAVI have also contributed to resource optimization. Non-anesthetized TAVI and other minimally invasive approaches could further reduce personnel and monitoring requirements.

While TAVI has shown dramatic resource savings, SAVR remains relatively constant in terms of hospital stays, ventilation times, and reimbursement costs despite new concepts of fast-track extubation after minimally invasive aortic valve replacement and early mobilization of patients. Here, the cost-effectiveness so far could not be proven and depends on the internal logistics of the hospitals. TAVI’s relative weight in the German DRG system is expected to continue declining, driven by ongoing medical and technological advancements. The procedure could eventually require fewer healthcare resources than SAVR, including significant reductions in hospital stays, ventilation support, and overall costs. Ambulatory TAVI, while speculative, represents the ultimate evolution of resource efficiency, positioning it far ahead of SAVR in terms of resource conservation.

Complications significantly prolong the length of hospital stay for both TAVI and SAVR. Historical data for TF-TAVI demonstrates a clear trend. The additional length of stay caused by a stroke increased from 3.03 days (2013) to 5.08 days (2021/2022) [10]. This rise is likely due to the shorter baseline hospital stay, which amplifies the relative impact of severe complications. Severe bleeding after TF-TAVI resulted in an additional 13 hospital days in both 2013 and 2021/2022 [10]. The extra length of stay of AKI after TF-TAVI decreased from 6.6 days (2013) to 5.28 days (2021/2022) [10], likely reflecting improvements in management strategies. The average extended stay after PPI in TF-TAVI dropped from 3.26 days (2013) to 2.54 days (2021/2022) [10]. Overall, even when complications like stroke, AKI, or PPI occur, the hospital stay following TAVI remains shorter than that after comparable complications in SAVR. Only in cases of severe bleeding hospital stays become similar for both procedures, but severe bleeding is less frequent in TF-TAVI (1.7% vs. 9.0%).

Ventilation duration is more strongly influenced by the type of complication. While PPI has minimal effect, complications like severe bleeding and AKI result in substantial increases in ventilation requirements.

The risk-adjusted additional reimbursement amounts for complications following TF-TAVI have changed significantly since 2013. The extra reimbursement for stroke decreased from 3,880 € (2013) to 2,039 € (2021/2022). The additional reimbursement for severe bleeding rose slightly from 12,604 € (2013) to 13,037 € (2021/2022). The extra reimbursement for AKI fell from 5,657 € (2013) to 2,153 € (2021/2022). Additional costs for PPI decreased from 646 € (2013) to 299 € (2021/2022) [10]. These numbers should be interpreted in the context of significant cost reductions for uncomplicated TAVI procedures since 2013, along with changes in the relative weight of DRGs for certain complications. These findings highlight the advantages of TAVI as a less invasive procedure, offering shorter recovery times, even when complications occur.

TAVI procedures, both with and without complications, tend to be more expensive than surgical aortic valve replacement (SAVR) in terms of total reimbursement volume. However, this cost discrepancy is expected to narrow in the future due to several factors. First, length of hospital stay is one of the primary cost drivers for any medical procedure, and TAVI already demonstrates shorter stays compared to SAVR, even in cases involving complications. Second, the costs of the prosthetic system (catheter and valve) are currently a significant expense in TAVI procedures. However, these costs are likely to decrease due to hospitals'stronger negotiating positions, larger purchase volumes, and increased competition among prosthesis manufacturers. An example of this trend is the prosthesis manufacturer Meril. In a clinical study, Meril's valve demonstrated non-inferiority to established TAVI prostheses [25], while offering competitive prices to hospitals. In contrast, there is currently no evidence to suggest a similar cost-reduction trajectory for SAVR. As a result, TAVI may increasingly establish itself as a more cost-effective alternative, particularly for high-risk or intermediate-risk patients.

### Study limitations

This study has several limitations. First, it is a retrospective study, so causal relationships cannot be proven with these data. Confounding factors beyond those examined in the study cannot be detected or controlled here. The risk adjustment performed cannot account for all potential confounding factors. The data are not randomized, which may lead to a bias in the results. Nevertheless, this study is based on nationwide"real-world"data from Germany, reflecting clinical practice. Therefore, any potential underreported bias observed and documented here also occurs in everyday practice across Germany. Due to the large dataset, potential biases may also have been leveled out. Risk adjustment was conducted to the extent possible with the available data source.

Furthermore, it cannot be ruled out that there may have been coding, billing, or data transmission errors, leading to over- or underdocumentation. Against this potential reliability limitation, it is argued that hospitals have no interest in undercoding, as their documentation is linked to their reimbursement. Over- or miscoding is checked randomly by the Medical Service of Health Insurance, providing a control mechanism.

The precision of the data is limited to the accuracy of the ICD and OPS codes. A deeper clinical characterization of patients, for example, through echocardiographic parameters or blood pressure, is not possible. It is also possible that the classification of heart failure according to NYHA for a patient may be outdated or imprecise. Finally, certain anatomical characteristics or measurements, such as body mass index, cannot be recorded.

The nationwide billing data from Germany are also not directly comparable with billing data from other countries and can only be directly compared with studies that also work with ICD and OPS codes. Similar inclusion criteria would be necessary for an adequate comparison of two studies based on data from the Federal Statistical Office.

The timing of complications is not recorded in the retrieved ICD and OPS codes, so it may occur that a complication arose before the heart valve procedure. This is unlikely, as heart valve operations are often planned procedures scheduled in advance and rarely follow other procedures.

Statements about procedures for pure aortic regurgitation, a related condition with similar surgical techniques, cannot necessarily be derived from these data.

The EuroSCORE, a preoperative risk parameter for surgical risk, was calculated as a"best case scenario"because not all parameters of the score were available. This results in a potentially lower surgical risk being assumed; however, this error affected both groups and likely did not significantly impact the risk-adjusted comparison.

Based on the patient characteristics, we see significant differences in the compared groups having significantly younger patients in the surgical arm with a different risk profile compared to the TAVI group. There will remain a group of patients that might benefit from either of the treatments in an equal way. In general, it is reasonable to assume that for a larger amount of patients one or the other treatment option will continue to be the gold standard of care. Our analysis is mostly important in the scenario of a patient group that is able to benefit from both treatments equally.

Additionally, data from the Federal Statistical Office can only capture events occurring during a hospital stay and do not provide long-term data and costs. Patients cannot be individually tracked. Individuals may appear twice in the database if they had two separate aortic valve procedures within the queried period.

Finally, no comparison between self-expanding and balloon-expandable TAVI prostheses was conducted here.

## Conclusions

With the continued progression of demographic change, an increase in the incidence of aortic valve stenosis is to be expected. A streamlined procedure with high medical efficacy, which ensures both low costs and high medical care quality, is therefore of high societal importance. The comparison of resource consumption with and without complications between two procedures for aortic valve stenosis treatment presented here is highly relevant. The two current treatment options differ significantly in their resource consumption, with surgical aortic valve replacement being more resource-intensive compared to the minimally invasive TF-TAVI. Nevertheless, TF-TAVI remains the more expensive procedure at present in Germany. Complications lead to increased healthcare resource consumption in both procedures, although the cost advantage of SAVR diminishes with complications, reaching comparable levels to TF-TAVI. Given the limited resources of payers, which must be sufficient for a growing number of patients and other costly innovative therapies, this comparative analysis can serve as a component in the evaluation of aortic valve stenosis treatment at the societal level.

## Funding/disclosures

AM was funded by the Berta-Ottenstein-Programme for Advanced Clinician Scientists, Faculty of Medicine, University of Freiburg. The authors declare that there is no conflict of interest. Open access funding enabled and organized by project DEAL.

## References

[1] Kazi, D.S., et al.: Forecasting the economic burden of cardiovascular disease and stroke in the United States through 2050: a presidential advisory from the American Heart Association. Circulation **150**, e89–e101 (2024) 38832515 10.1161/CIR.0000000000001258

[2] Coffey, S., et al.: Global epidemiology of valvular heart disease. Nat. Rev. Cardiol. **18**, 853–864 (2021) 34172950 10.1038/s41569-021-00570-z

[3] Kermanshahchi, J., et al.: A review of the cost effectiveness of transcatheter aortic valve replacement (TAVR) versus surgical aortic valve replacement (SAVR). Cureus **15**, e46535 (2023) 37927639 10.7759/cureus.46535 PMC10625447

[4] Kermanshahchi, J., et al.: Transcatheter aortic valve replacement (TAVR) versus surgical aortic valve replacement (SAVR): a review on the length of stay, cost, comorbidities, and procedural complications. Cureus **16**, e54435 (2024) 38510891 10.7759/cureus.54435 PMC10951673

[5] Vahanian, A., et al.: 2021 ESC/EACTS guidelines for the management of valvular heart disease. Eur. Heart J. **43**, 561–632 (2022) 34453165 10.1093/eurheartj/ehab395

[6] Reynolds, M.R., et al.: Cost-effectiveness of transcatheter aortic valve replacement compared with surgical aortic valve replacement in high-risk patients with severe aortic stenosis: results of the PARTNER (Placement of Aortic Transcatheter Valves) trial (Cohort A). J. Am. Coll. Cardiol. **60**, 2683–2692 (2012) 23122802 10.1016/j.jacc.2012.09.018

[7] Reynolds, M.R., et al.: Cost-effectiveness of transcatheter aortic valve replacement with a self-expanding prosthesis versus surgical aortic valve replacement. J. Am. Coll. Cardiol. **67**, 29–38 (2016) 26764063 10.1016/j.jacc.2015.10.046 PMC4959424

[8] Galper, B.Z., Baron, S.J., Cohen, D.J.: Cost-effectiveness of TAVR in low-risk patients: do we have more than a NOTION? EuroIntervention **15**, e953–e955 (2019) 31806585 10.4244/EIJV15I11A179

[9] Baron, S.J., Ryan, M.P., Moore, K.A., Clancy, S.J., Gunnarsson, C.L.: Contemporary costs associated with transcatheter versus surgical aortic valve replacement in Medicare beneficiaries. Circ. Cardiovasc. Interv. **15**, e011295 (2022) 35193382 10.1161/CIRCINTERVENTIONS.121.011295

[10] Kaier, K., et al.: The impact of post-procedural complications on reimbursement, length of stay and mechanical ventilation among patients undergoing transcatheter aortic valve implantation in Germany. Eur. J. Health Econ. **19**, 223–228 (2018) 28229254 10.1007/s10198-017-0877-7

[11] Grube, E., Sinning, J.-M.: The ‘Big Five’ Complications After Transcatheter Aortic Valve Replacement: Do We Still Have to Be Afraid of Them? JACC Cardiovasc. Interv. **12**, 370–372 (2019) 30784642 10.1016/j.jcin.2018.12.019

[12] Reinöhl, J., et al.: Effect of availability of transcatheter aortic-valve replacement on clinical practice. N. Engl. J. Med **373**, 2438–2447 (2015) 26672846 10.1056/NEJMoa1500893

[13] Oettinger, V., et al.: Comparing balloon-expandable and self-expanding transfemoral transcatheter aortic valve replacement based on subgroups in Germany 2019/2020. Clin. Res. Cardiol. **113**, 168–176 (2024) 37982864 10.1007/s00392-023-02326-w PMC10808194

[14] Maier, A., et al.: Procedural safety of rotational atherectomy and modified balloon angioplasty: insights from a German national registry. Clin. Res. Cardiol. (2024). 10.1007/s00392-024-02538-8 39259363 10.1007/s00392-024-02538-8 PMC12783188

[15] Maier, A., et al.: Catheter based left atrial appendage closure in-hospital outcomes in Germany from 2016 to 2020. Clin. Res. Cardiol. **113**, 1419–1429 (2024) 37698619 10.1007/s00392-023-02299-w PMC11420385

[16] Kaier, K., et al.: Estimating the additional costs per life saved due to transcatheter aortic valve replacement: a secondary data analysis of electronic health records in Germany. Eur. J. Health Econ. **20**, 625–632 (2019) 30600467 10.1007/s10198-018-1023-x

[17] Mack, M.J., et al.: Transcatheter aortic-valve replacement in low-risk patients at five years. N. Engl. J. Med. **389**, 1949–1960 (2023) 37874020 10.1056/NEJMoa2307447

[18] Stachon, P., et al.: Transapical aortic valve replacement versus surgical aortic valve replacement: A subgroup analyses for at-risk populations. The Journal of Thoracic and Cardiovascular Surgery **162**, 1701-1709.e1 (2021) 32222407 10.1016/j.jtcvs.2020.02.078

[19] Kaier, K., et al.: Sex-specific differences in outcome of transcatheter or surgical aortic valve replacement. Can. J. Cardiol. **34**, 992–998 (2018) 30056851 10.1016/j.cjca.2018.04.009

[20] GKV-Spitzenverband. Bundesbasisfallwert (BBFW). https://www.gkv-spitzenverband.de/krankenversicherung/krankenhaeuser/budgetverhandlungen/bundesbasisfallwert/bundesbasisfallwert.jsp. Accessed 10 Sept 2025

[21] DRG F98B | Erlös und Pflegeentgelt | Gebietsanalysen | CMI. https://app.reimbursement.info/drgs/F98B?year=2022. Accessed 10 Sept 2025

[22] DRG F98B | Erlös und Pflegeentgelt | Gebietsanalysen | CMI. https://app.reimbursement.info/drgs/F98B?year=2014. Accessed 10 Sept 2025

[23] DRG F03D | Erlös und Pflegeentgelt | Gebietsanalysen | CMI. https://app.reimbursement.info/drgs/F03D?year=2014. Accessed 10 Sept 2025

[24] DRG F03D | Erlös und Pflegeentgelt | Gebietsanalysen | CMI. https://app.reimbursement.info/drgs/F03D?year=2022. Accessed 10 Sept 2025

[25] Baumbach, A., et al.: Landmark comparison of early outcomes of newer-generation Myval transcatheter heart valve series with contemporary valves (Sapien and Evolut) in real-world individuals with severe symptomatic native aortic stenosis: a randomised non-inferiority trial. Lancet **403**, 2695–2708 (2024) 38795719 10.1016/S0140-6736(24)00821-3

